# Acute stress may induce ovulation in women

**DOI:** 10.1186/1477-7827-8-53

**Published:** 2010-05-26

**Authors:** Juan J Tarín, Toshio Hamatani, Antonio Cano

**Affiliations:** 1 Department of Functional Biology and Physical Anthropology, Faculty of Biological Sciences, University of Valencia, Burjassot, Valencia 46100, Spain; 2 Department of Obstetrics and Gynecology, Keio University School of Medicine, Tokyo 160-8582, Japan; 3 Department of Pediatrics, Obstetrics and Gynecology, Faculty of Medicine, University of Valencia, Valencia 46010, Spain

## Abstract

**Background:**

This study aims to gather information either supporting or rejecting the hypothesis that acute stress may induce ovulation in women. The formulation of this hypothesis is based on 2 facts: 1) estrogen-primed postmenopausal or ovariectomized women display an adrenal-progesterone-induced ovulatory-like luteinizing hormone (LH) surge in response to exogenous adrenocorticotropic hormone (ACTH) administration; and 2) women display multiple follicular waves during an interovulatory interval, and likely during pregnancy and lactation. Thus, acute stress may induce ovulation in women displaying appropriate serum levels of estradiol and one or more follicles large enough to respond to a non-midcycle LH surge.

**Methods:**

A literature search using the PubMed database was performed to identify articles up to January 2010 focusing mainly on women as well as on rats and rhesus monkeys as animal models of interaction between the hypothalamic-pituitary-adrenal (HPA) and hypothalamic-pituitary-gonadal (HPG) axes.

**Results:**

Whereas the HPA axis exhibits positive responses in practically all phases of the ovarian cycle, acute-stress-induced release of LH is found under relatively high plasma levels of estradiol. However, there are studies suggesting that several types of acute stress may exert different effects on pituitary LH release and the steroid environment may modulate in a different way (inhibiting or stimulating) the pattern of response of the HPG axis elicited by acute stressors.

**Conclusion:**

Women may be induced to ovulate at any point of the menstrual cycle or even during periods of amenorrhea associated with pregnancy and lactation if exposed to an appropriate acute stressor under a right estradiol environment.

## Background

It is known that the percentage of pregnancies resulting from single episodes of forced penile-vaginal intercourse (rape) is significantly higher (8.0% in a sample of 405 women from a national random-digit dialing sample of households in USA) than the percentage of pregnancies resulting from single episodes of consensual, unprotected intercourse (3.1% in a sample of 221 women with no fertility problems planning to become pregnant in USA) [1]. It is worth mentioning that data from the study by Gottschall and Gottschall [1] were adjusted for the use of oral contraception and intra-uterine devices (IUDs). Furthermore, Gottschall and Gottschall [1] elegantly ruled out the possibility that in their study higher rape-pregnancy rates may result from (1) women being more likely to report to medical or law-enforcement authorities rapes resulting in conception; (2) women sometimes attributing paternity to rapists when they were fertilized by a consensual partner; and (3) a high number of rape victims coming from the most fecund age cohorts of the population, i.e. rapists disproportionately would target young women in their most fecund years. These points were the main grounds used in previous studies to reject the fact that per-incident rape-pregnancy risk had been reiteratively reported to be higher than per-incident consensual pregnancy risk.

It is also known that, in fertile women planning to become pregnant, ovulation and conception may occur on any day of the menstrual cycle, although the maximum probability is reached at the middle of the cycle [2]. Furthermore, ovulations and conceptions may arise during periods of amenorrhea associated with oral contraceptive use, drug addiction, pregnancy (superfetation; for review, see Pape et al. [3]) and lactation. In addition, fertilization of ≥ 2 oocytes from the same menstrual cycle by sperm from separate acts of sexual intercourse has been also reported (superfecundation [4,5]).

All this epidemiological evidence, together with the fact that copulation can trigger or hasten (facilitate) ovulation in otherwise spontaneous ovulating species such as the rat (for reviews, see Gibson et al. [6], Milligan [7], Bakker and Baum [8] and Nagy et al. [9]), led Zarrow et al. [10] and Jöchle [11,12] to propose that women may be facultative coitus-induced ovulators.

This attractive and stimulating hypothesis is supported by the fact that 68% of women display 2 follicular waves and the remaining 32% exhibit 3 waves of ovarian follicular development during an interovulatory interval [13]. It is likely that women exhibit waves of follicular development during pregnancy and lactation as it occurs in cattle, sheep, goats and mares [14] (for review, see Evans [15]).

The presence of multiple follicular waves in women may provide an extra source of oocytes to be ovulated if an appropriate non-midcycle luteinizing hormone (LH) surge took place. We should note that follicular waves can be either ovulatory (the final wave of follicular development) or anovulatory (all the preceding waves) [13,16]. Twenty one percent of the anovulatory follicular waves are major, i.e. those in which one follicle is selected to be dominant over other follicles of the wave, and the remaining 79% minor, i.e. those in which no selection of a dominant follicle is evidenced. Although, anovulatory follicles do not develop as large as ovulatory follicles, they grow to an ostensible preovulatory diameter before undergoing atresia (maximum diameter of the dominant follicle of an anovulatory major wave: 12.9 ± 0.8 mm, range 10-17 mm; maximum diameter of the largest follicle of an anovulatory minor wave: 8.1 ± 0.1 mm, range 7-9 mm) [17]. The maximum diameters reached by dominant follicles from anovulatory major waves (range 10-17 mm) are compatible with potential ovulations if follicles are correctly stimulated. In fact, spontaneous ovulations in normal menstrual cycles have been reported to occur in follicles ≥ 15 mm in diameter [16].

Despite the fact that women display multiple waves of follicular development during an interovulatory interval, and likely during pregnancy and lactation, the hypothesis that women may be coitus-induced ovulators is directly refuted by studies showing that neither coitus nor orgasm induces a surge in LH secretion (and thus ovulation) in women [18-20] and rhesus monkeys [21]. Results from these classical studies have been recently endorsed by Baerwald et al. [13,16]. These authors tracked daily the follicle diameter and follicle number present in the ovaries of 50 healthy women of reproductive age (28.0 ± 6.9, range 19-43 years) displaying clinically normal menstrual cycles and not taking medications known to interfere with reproductive function. The ovarian ultrasonographic examinations only evidenced ovulations in follicles from the last wave of the interovulatory cycle, which emerges at the early follicular phase. The preceding waves, emerging at the luteal phase, were anovulatory in all 50 women entered into the study. This fact contradicts the assumption that coitus may induce ovulation in women. We should bear in mind that, in addition to a periovulatory peak, human beings display no changes at all or even rises in male- and female-initiated sexual activity, woman's sexual desire, autosexual activity and sexual arousability, and interpersonal sexual activities, including sexual intercourse, during the mid-follicular and late-luteal phases (for review, see Tarín and Gómez-Piquer [22]). Thus, it is expected that a non-negligible number of the 50 women analyzed by Baerwald et al. [13,16] was presumably engaged in sexual intercourse during the period of ultrasonographic evaluations (one interovulatory cycle).

If we consider the laboratory rat as a paradigm of facultative coitus-induced ovulation, it can be noted that there are notable differences between women and rats in the neuroendocrine mechanisms controlling the ovarian cycle. For instance, in contrast to women, both spontaneous and induced ovulatory mechanisms are integrated in the rat. Female rats have no functional corpora lutea and must receive vulval, vaginal and/or cervical intromissive stimulation in order for the ovaries to develop fully functional corpora lutea. Mating stimulates the release of prolactin from the anterior pituitary, which is required for activation of the corpora lutea and progesterone biosynthesis (for review, see Bakker and Baum [8]). Furthermore, although technical difficulties have precluded determining the ovarian dynamics in rats by transcutaneous ultrasound bio-microscopy [23], it is expected that rats exhibit a single wave of follicular development such as evidenced in the mouse [23] and the coitus-induced ovulating species analyzed as of today, including cats, llamas and camels (for review, see Evans [15]).

### Interaction of the hypothalamic-pituitary-adrenal (HPA) and hypothalamic-pituitary-gonadal (HPG) axes

While the woman's reproductive physiology does not fit the rat paradigm of facultative coitus-induced ovulation, the HPA and HPG axes interact with each other in a similar way in both species. Indeed, in rats and women, there is a positive coupling between the HPA axis and the HPG axis at the time of the preovulatory LH surge. In women, initiation of the LH surge takes place either at 04.00 a.m. in 20% of the cases or at 08.00 a.m. in the remaining 80% of the cases. The onset of the LH surge is strongly coupled with the time at which the peak (acrophase) of the cortisol circadian rhythm occurs, i.e. maximal cortisol plasma concentrations take place at 04.00 a.m. when the LH preovulatory surge initiates at 04.00 a.m. and at 08.00 a.m. when the LH preovulatory surge starts at 08.00 a.m. [24]. Likewise, in the laboratory rat, both the preovulatory LH surge and the maximal plasma values of the corticosterone circadian rhythm are observed during the transition from the light to the dark phase (2 h preceding and 2 h following) on the day of proestrus [25,26].

Moreover, it is well-known that prolonged or chronic stress in rats and women may block, inhibit or delay the preovulatory LH surge and therefore disrupt the estrous or menstrual cycle (for reviews, see Rivier and Rivest [27] and Kalantaridou et al. [28]). On the contrary, women and rats exposed to acute stress may respond with an adrenal-progesterone-induced LH surge (for review, see Mahesh and Brann [29]).

There is considerable evidence in rats and women showing an elevation in the levels of serum progesterone mainly from the adrenal glands prior to the onset of the LH surge. This increase in progesterone levels serves to initiate, synchronize, potentiate and limit the preovulatory LH surge to a single day (for review, see Mahesh and Brann [29]). In order for progesterone to exert its facilitatory role on gonadotropin secretion, the presence of a background of high estradiol is essential [30]. This is due to the fact that estradiol induces the expression of anterior pituitary, hypothalamic and extrahypothalamic progesterone receptors, which function as transcriptional regulators that prompt alterations in gene expression needed for facilitation of neurosecretion of gonadotropin-releasing hormone (GnRH) surges and release of periovulatory gonadotropin surges (for reviews, see Mahesh and Brann [29] and Levine et al. [31]).

It is not surprising, therefore, that progesterone and natural mineralocorticoids, such as deoxycorticosterone, and synthetic glucocorticoids, such as triamcinolone acetonide, which possess "progestin-like" activity, stimulate the release of LH and follicle-stimulating hormone (FSH) when administered acutely to pregnant mare serum gonadotropin (PMSG)-primed immature [32] or estrogen-primed ovariectomized immature rats [33]. In estrogen-treated menopausal women, it has been demonstrated that progesterone administration is able to induce a pre-ovulatory-type surge of LH and FSH (for review, see Mahesh and Brann [29]. These findings have led to propose that the improvement in menstrual rhythm and ovulatory activity following glucocorticoid therapy in women suffering from polycystic ovarian syndrome or other syndromes of androgen excess may be due to its direct effects on the release of gonadotropins, in addition to the ability of glucocorticoids to suppress adrenal overproduction of androgens [32,33].

Moreover, it has been reported that a single injection of adrenocorticotropic hormone (ACTH) to estrogen-primed intact and ovariectomized immature rats causes a significant elevation in serum LH and FSH levels. However, this ACTH treatment fails to induce a surge of gonadotropins in non-estrogen-primed intact immature rats. It is worth mentioning that of the 2 major adrenal steroids secreted as result of ACTH administration, i.e. progesterone and corticosterone, only progesterone is able to stimulate LH release in estrogen-primed ovariectomized immature rats on the day of its administration [34]. Likewise, a 3-h intravenous infusion of ACTH to postmenopausal or ovariectomized women with estrogen replacement raises the plasma levels of corticosterone and progesterone 3-4 h after injection, accompanied by a significant stimulation of LH release 2-3 h after the initial raise in progesterone [35].

### Aim of the study

The aim of this bioessay is to gather information either supporting or rejecting the hypothesis that acute stress may induce ovulation in women. The formulation of this hypothesis is based on 2 facts: 1) estrogen-primed postmenopausal or ovariectomized women display an adrenal-progesterone-induced ovulatory-like LH surge in response to exogenous ACTH administration; and 2) women display multiple follicular waves during an interovulatory interval, and likely during pregnancy and lactation. Thus, acute stress may induce ovulation in women displaying appropriate serum levels of estradiol and one or more follicles large enough to respond to a non-midcycle LH surge.

## Methods

As the HPA and HPG axes interact with each other in a similar way in rats and women, a literature search using the PubMed database [36] was performed to identify all the articles up to January 2010 dealing with the response of the HPA and HPG axes to acute stressors in rats and women. Studies on rhesus monkeys (*Macaca mulatta*) and previous reviews were also consulted. The following key words were used: "acute stress", "gonadotropin secretion", "luteinizing hormone surge", "hypothalamic-pituitary-adrenal axis", "hypothalamic-pituitary-gonadal axis", "premature luteinization" and "adrenal progesterone secretion".

### Changes in serum levels of estradiol during the ovarian cycle in rats and women

As the serum concentration of estradiol in rats and women fluctuates during the estrous or menstrual cycle, it is expected that the LH-release response of the anterior pituitary to an acute-stress-induced surge of adrenal progesterone will vary during the estrous or menstrual cycle. Thus, in order to analyze the studies reviewed in this bioessay, it is important to recall the pattern of fluctuations in serum levels of estradiol during the ovarian cycle in both rats and women (female macaque monkeys have a pattern of estradiol variation similar to women [37]). In particular, in Fischer 344 rats, serum levels of estradiol display one single cycle of increasing and decreasing levels during the 4- or 5-day estrous cycles. Serum concentration of estradiol shows a single peak of ≈ 257 pmol/L on the morning of proestrus, preceding the zenith of the LH surge (≈ 0.076 IU/L) that arises during the transition from the light to the dark phase. The peak of estradiol is followed by a nadir of ≈ 55 pmol/L on the night of proestrus, with this baseline level being kept during estrus and diestrus-1. Finally, on the morning of diestrus-2, estradiol levels start to rise, reaching a level of ≈ 165 pmol/L on the night of diestrus-2, and a level of ≈ 239 pmol/L on the night of diestrus-3 in 5-day cyclers [26].

On the other hand, in women, serum concentration of estradiol shows 2 cycles of increasing and decreasing levels. Serum baseline levels of 147-165 pmol/L estradiol start to increase ≈ 5 or 6 days before the preovulatory peak of LH in women with 3 or 2 follicular waves, respectively. It reaches a maximum level of ≈ 808 pmol/L 1 day before the preovulatory peak of LH (≈ 26 IU/L) in women with 2 follicular waves, or coinciding with the peak of LH (≈ 35 IU/L) in women with 3 follicular waves. After reaching this maximum, serum levels of estradiol drop to the baseline level of 147-165 pmol/L on day 3 after ovulation but elevate thereafter to ≈ 367 pmol/L for a week in women with 2 or 3 follicular waves, although women with 3 follicular waves exhibit more fluctuating values. At the end of the luteal phase, serum concentration of estradiol drops to the baseline level of 147-165 pmol/L, with this level being kept steady during the early follicular phase [13].

## HPA axis response to acute stress

### Effect of ovarian cycle

Table 1 shows the effect of ovarian cycle on HPA axis response to acute stress in rats, rhesus monkeys and women.

**Table 1:** Effect of ovarian cycle on HPA axis response to acute stress.

Species	Stressor	Phase of the cycle	HPA axis response	References
Rats	Surgery	On the morning of proestrus	Positive	[38]
	20-min restraint	On the morning of proestrus vs either estrus or diestrus	Increased	[39]
		Proestrus, estrus and diestrus II	No cycle effect	[40]
Rhesus monkeys	30-min intracerebroventricular administration of interleukin-1α	Mid-follicular vs early-follicular phase	Increased	[41]
Women	Bilateral ovariectomy plus total hysterectomy	Mid- to late-follicular phase	Positive	[43]
		Early- to mid-luteal phase	No response	[42,43]
	Cholecystectomy	Early- to mid-follicular phase	Positive	[42]
	20-min progressive submaximal treadmill exercise	Mid-luteal phase vs early follicular phase	Increased	[44]
	90-min submaximal bicycle exercise	Mid-luteal phase vs mid-follicular phase	Increased	[46]
	90-min submaximal treadmill exercise	Mid-luteal phase vs early- and late-follicular phase	Increased	[47]
	60-min progressive submaximal treadmill exercise	Mid-follicular and luteal phase	No cycle effect	[49]
	Progressive maximal treadmill exercise to voluntary exhaustion or 40-min submaximal treadmill exercise	Early-follicular vs mid-luteal phase	No cycle effect	[50]
	20-min progressive submaximal aerobic treadmill	Early-mid-follicular, periovulatory and mid-late luteal phase	No cycle effect	[51]
	Psychological stress of remembering stressful situations in their lives and self-evaluation	Menstrual and periovulatory (late-follicular, ovulatory and early-luteal phases) phase	No cycle effect	[52]
	Psychological stress of self-evaluation	Ovulatory period vs premenstrual phase	Decreased	[48]

#### Rats

In rats with 5-day estrous cycles, the surgical stress of laparotomy on the morning of proestrus leads to release of adrenal estradiol and progesterone preceding an early surge of LH from the pituitary [38]. Moreover, intact 4- and 5-day cycling rats exhibit increased HPA sensitivity, releasing higher amounts of ACTH and corticosterone into the blood stream, immediately after a 20-min restraint on the morning of proestrus than animals at either estrus or diestrus (combined diestrus-1, 2 and 3) [39]. Notwithstanding, Carey et al. [40] found higher plasma levels of ACTH and corticosterone immediately after a 20-min restraint in 4-day cycling rats but did not evidence a significant effect of the estrous cycle on plasma concentrations of these hormones

#### Non-human primates

In cycling female rhesus monkeys, a 30-min intracerebroventricular administration of interleukin-1α, which simulates an inflammatory/immune-like challenge, induces a higher increase in plasma levels of cortisol and progesterone if administered during the mid-follicular phase compared to the early-follicular phase [41].

#### Women

Women with normal cycles undergoing bilateral ovariectomy plus total hysterectomy under general anesthesia during the mid- to late-follicular phase of the menstrual cycle respond with a small increase in plasma levels of progesterone 12 h after surgery preceding a small rise of LH. Likewise, after the surgical stress of cholecystectomy for benign conditions of the gallbladder under general anesthesia during the early- to mid-follicular phase, women exhibit a small surge-like increase in plasma levels of progesterone 12 h after the operation [42]. These increases, however, are not observed in women undergoing bilateral ovariectomy plus total hysterectomy during the early- to mid-luteal phase [42,43].

Other studies show that women in the mid-luteal phase of the menstrual cycle display enhanced plasma levels of ACTH in response to a 20-min progressive treadmill exercise compared to women in the early follicular phase [44]. Mid-luteal phase women also display reduced sensitivity to glucocorticoid feedback (lower suppression of plasma cortisol in response to a low dose of synthetic glucocorticoid dexamethasone) and decreased glucocorticoid receptor (type II) mRNA expression in lymphocytes compared to women in the early follicular phase [45]. In addition, the cortisol response to a 90-min submaximal bicycle [46] or treadmill [47] exercise is higher in the mid-luteal phase than in the early-follicular [47], mid-follicular [46] and late-follicular phases [47]. Likewise, a lower adrenocortical reactivity (decreased plasma cortisol levels) to the psychological stress of self-evaluation has been evidenced during the ovulatory period (between 12-16 days from the onset of the previous menstruation) than during the premenstrual phase (between 1-3 days prior to the onset of the next menstruation) [48]. There are, however, other studies that either do not find menstrual cycle-related differences in the HPA axis responsivity to intense and moderate physical exercise [49-51] or to the psychological stress of remembering stressful situations in their lives and self-evaluation [52]. Interestingly, although Galliven et al. [51] did not find a significant effect of the menstrual cycle on plasma cortisol levels after a 20-min progressive submaximal treadmill, the analysis of the net integrated area under the curve revealed a marginal (P = 0.056) lower response of cortisol in the periovulatory (between 10-16 days after the start of menses) phase compared with the early-mid-follicular (between 3-9 days after the start of menses) and mid-late-luteal (between 18-26 days after the start of menses) phases.

#### Concluding remarks

From the studies analyzed in this section, it seems that the ovarian cycle modulates the HPA axis response to acute stressors. In rats, it appears that proestrus morning is the more sensitive period. In rhesus monkeys, the HPA axis response is higher during the mid-follicular phase than during the early-follicular phase. And in women, the period more responsive seems to be during the mid-follicular and mid- and late-luteal phases. The absence of effect of the menstrual cycle on HPA axis responsivity to physical stress found in several studies may be explained by the presence of uncontrolled variables related to the training status of women and possibly to the training regimen followed in the days previous to the exercise test (cited by Williams et al. [53]).

### Effect of hormone treatment

Table 2 shows the effect of hormone treatment on HPA axis response to acute stress in rats, rhesus monkeys and women.

**Table 2:** Effect of hormone treatment on HPA axis response to acute stress.

Species	Stressor	Hormone treatment	HPA axis response	References
Rats	20-min restraint	Estradiol vs oil and estradiol plus progesterone	Increased	[39]
	Exposition to a novel environment	Estradiol and estradiol plus progesterone vs no hormone treatment and progesterone	Increased	[40]
	5-sec footshock	Estradiol vs no hormone treatment	Increased	[54]
	1-min exposure to ether vapors	Estradiol vs no hormone treatment	Increased	[54]
Rhesus monkeys	30-min intracerebroventricular infusion of interleukin-1α	Estradiol at doses resulting in the typical high levels of plasma estradiol that reproduce the late follicular phase	Increased	[56]
		Estradiol at doses resulting in low levels of plasma estradiol	Increased	[57]
		Estradiol at doses that results in intermediate levels of plasma estradiol that reproduce the early-mid follicular phase	Inhibited	[57]
Women	Single intravenous injection of endotoxin	Estradiol vs no hormone treatment	No treatment effect	[35]
	Psychological stress (speech and math tasks)	Estrogens or estrogens plus progestogen vs no hormone treatment	No treatment effect	[58]
		Estradiol vs the same women before treatment	Decreased	[59]
	Psychological stress (mental arithmetic tasks accompanied by a repetitive annoying background noise	Estradiol vs placebo	Decreased	[60]

#### Rats

It is known that immediately after exposure of ovariectomized rats to a 20-min restraint stress the plasma levels of ACTH are higher in estradiol-treated rats compared to the oil-treated control and to estradiol plus progesterone-treated rats. Moreover, during the 20-min period of restraint, the plasma levels of ACTH and corticosterone are always higher in the estradiol-treated group than in the oil-treated control and estradiol plus progesterone-treated rats [39]. Likewise, during the first 60 min after exposure to stress from a novel environment, ovariectomized estradiol- and estradiol plus progesterone-treated rats display an enhanced ACTH response compared to ovariectomized non-treated control and progesterone-treated rats. Ovariectomized estradiol plus progesterone-treated rats also display a higher corticosterone response than non-treated control and progesterone-treated rats during the same period of time [40].

Plasma levels of ACTH and corticosterone during the 2 h after a 5-sec footshock are higher in ovariectomized estradiol-treated rats than in ovariectomized non-treated control rats. At 20 min after 1-min exposure to ether vapors, plasma concentration of corticosterone is also higher in the group of estradiol-treated rats. Moreover, the ACTH and corticosterone secretory responses are less effectively suppressed by RU 28362 (a specific glucocorticoid receptor agonist) in estradiol-treated animals than in non-treated control rats [54]. This finding endorses other studies showing an inhibitory effect of estradiol on glucocorticoid receptor-mediated negative feedback [55].

#### Non-human primates

Studies in ovariectomized estrogen-replaced rhesus monkeys show that ACTH, cortisol and progesterone responses to a 30-min intracerebroventricular infusion of interleukin-1α are increased compared to the responses found in ovariectomized estrogen-replaced control monkeys infused intracerebroventricularly with a physiological saline solution. Experimental females received estrogen replacement therapy during 5 days at doses that elevated plasma estradiol concentration to 378 pmol/L (level that reproduces the typical high estradiol concentrations of the late follicular phase) [56]. The cortisol response to this immune-inflammatory challenge is also increased in ovariectomized 5-day-estrogen-treated monkeys with plasma concentrations of estradiol ≤ 73 pmol/L [57]. However, in 5-day-replaced rhesus monkeys with plasma levels of 114 pmol/L estradiol (concentration similar to that of the early-mid follicular phase), the cortisol response is inhibited [57].

#### Women

Studies in estrogen-replaced menopausal or ovariectomized women provide intriguing results. Some reports evidence no effect of estradiol treatment on (1) the increases of plasma cortisol and progesterone observed in response to a single intravenous injection of endotoxin after 4 weeks of treatment [mean ( ± SEM) plasma estradiol levels: 23.5 ± 3.3 pmol/L in unreplaced women and 315.7 ± 36.7 pmol/L after estradiol replacement] [35]; or (2) the cortisol response to psychological stressors (speech and math tasks) after at least 2 years of estrogen alone (a mixture of 6 estrogenic substances) or estrogen and progestogen treatment (authors did not specify the plasma levels of estradiol of replaced and unreplaced women) [58]. Another study shows no changes in plasma ACTH concentration or even decreased peak levels of plasma cortisol and androstenedione in response to psychological stressors (speech and math tasks) after 6 weeks of treatment with estradiol compared to the increases evidenced in these women before estradiol replacement (plasma estradiol levels: ≤ 73.4 pmol/L before estradiol replacement and 587.4 ± 58.7 pmol/L after estradiol replacement) [59]. In perimenopausal women (within 2 years of their last period and actively experiencing vasomotor symptoms of menopause), estradiol supplementation for 8 weeks results in decreased plasma levels of ACTH and cortisol after a psychological stress (mental arithmetic tasks accompanied by a repetitive annoying background noise to induce difficulty in concentration) compared to placebo-treated perimenopausal women (plasma estradiol levels: 115.0 ± 19.0 pmol/L in placebo-treated women and 1118.0 ± 111.0 pmol/L in estradiol-treated women) [60].

As far as we know, there are no studies reporting increases in HPA axis reactivity to acute stressors in estrogen-treated perimenopausal, menopausal or ovariectomized women. Only one study in young men has found increased peak ACTH and cortisol responses to a brief psychosocial stress (free speech and mental arithmetic in front of an audience) after 24-48 h of estradiol treatment compared to a placebo group [61].

#### Concluding remarks

The studies focused on the effects of hormone replacement on HPA axis response suggest that estrogen treatment in ovariectomized females or perimenopausal and menopausal women may modify the HPA axis response to acute stressors. In rats, it appears that estradiol treatment increases the responsivity of the HPA axis to different acute stressors. In rhesus monkeys, the HPA axis response to a 30-min intracerebroventricular infusion of interleukin-1α depends on the plasma level of estradiol after estrogen replacement. In particular, low and high plasma concentrations increase the response whereas intermediate concentrations are inhibitory. In contrast, estrogen replacement in women does not change or even may decrease the typical response of the HPA axis to acute stressors. Although it is difficult to compare among studies the absolute plasma levels of estradiol exhibited by women before being exposed to acute stressors because different measurement methods were used, it is worth mentioning that, in all the studies analyzed in this section, independently of the replacement regimen followed, the HPA axis always displayed a positive response when plasma levels of estradiol were relatively low (≤ 73.4 pmol/L) or intermediate (115.0-315.7 pmol/L) whereas the response was decreased when plasma levels of estradiol were relatively high (587.4-1118.0 pmol/L). Thus, it can be hypothesized that the response of the HPA axis to inflammatory or psychological stressors evidenced in perimenopausal, menopausal or ovariectomized women may depend on the range of plasma estradiol concentration present in women when exposed to these stressors.

## Acute-stress-induced LH release

### Effect of ovarian cycle

Table 3 shows the effect of ovarian cycle on acute-stress-induced LH release in rats, rhesus monkeys and women.

**Table 3:** Effect of ovarian cycle on acute-stress-induced LH release.

Species	Stressor	Phase of the cycle	LH release	References
Rats	Placed in a mating cage without a male or laparotomy	Persistent-vaginal-estrus (estradiol levels similar to the levels on the morning of proestrus)	Positive (inferred by ovulation)	[63]
	Laparotomy	On the morning of proestrus	Advance of the time of the primary LH surge	[38]
		On the morning of diestrus-2	Delay of the time of the primary LH surge (and ovulation)	[64]
	Intracerebroventricular injection of interleukin-1α or β	On the morning of proestrus	Inhibition of the primary LH surge (and ovulation)	[65]
	Rapid blood volume depletion	At diestrus (combined diestrus-1 and 2)	Positive	[66]
Rhesus monkeys	30-min chair restraint	Mid-follicular and mid-luteal phases	Positive	[67]
	30-min intracerebroventricular administration of interleukin-1α	Mid-follicular phase	Positive	[41]
		Early follicular phase	No release	[41]
Women	Bilateral ovariectomy plus total hysterectomy	Mid- to late-follicular phase	Positive	[43]
		Early- to mid-luteal phase	No release	[43]
	Cholecystectomy	Early- to mid-follicular	Positive	[42]
	Progressive submaximal treadmill exercise to exhaustion	Mid-follicular and mid-luteal phase	No release	[69]
	90-min submaximal bicycle exercise	Mid-follicular and mid-luteal phase	Decreased plasma levels of LH	[46]
	60-min progressive submaximal treadmill exercise	Mid-follicular phase	Positive	[49]
		Mid-follicular phase	Positive	[53]
		Mid-luteal phase	No release	[70]

#### Rats

Brown-Grant et al. [62] reported that ≈ 50% of light-induced persistent-vaginal-estrus rats, which show elevated levels of estradiol (similar to the levels on the morning of proestrus in rats maintained under a controlled photoperiod [63]), can ovulate when placed in a mating cage without a male or when subjected to a more severe stress such as laparotomy. The laparotomy-associated stress can also advance the time of LH surge (zenith: ≈ 0.130 IU/L) in 5-day cycling rats if performed on the morning of proestrus [38]. On the contrary, in 4-day cyclers, laparotomy delays pituitary LH discharge and ovulation if carried out on the morning of diestrus-2 [64]. An inhibition of the primary LH surge and ovulation has also been found in 4-day cycling rats after intracerebroventricular injection of interleukin-1α or β on the morning of proestrus [65]. Furthermore, it has been reported that a rapid blood volume depletion from the external jugular veins under constant ether anesthesia is able to increase plasma LH concentrations (from ≈ 0.017 IU/L to 0.043 IU/L) during the first 10 min after the initial blood loss in 4-day cycling rats at diestrus (combined diestrus-1 and 2) [66].

#### Non-human primates

In cycling rhesus monkeys, Norman et al. [67] found that a prolonged (6 h) chair restraint resulted in suppression of LH release, but 1 out of the 9 females tested exhibited a 2-5-fold increase in plasma levels of LH within 30 min of the initiation of restraint in both the mid-follicular and mid-luteal phases, as well as an increase in frequency of LH pulses over that normally evidenced in the mid-luteal phase. Furthermore, a 30-min intracerebroventricular administration of interleukin-1α induces a 3-fold increase in LH by 5 h after interleukin-1α infusion if administered during the mid-follicular phase, but there is no LH response during the early follicular phase [41]. We should mention that 1 out of the 9 mid-follicular monkeys exhibited a sustained surge-like release of LH after interleukin-1α treatment reaching a maximum (≈ 0.130 IU/L) at 13 h after treatment [41]. These findings contrast with the inhibition of the primary LH surge in cycling rats after intracerebroventricular injection of interleukin-1α or β on the morning of proestrus (see above). Discrepancies between rhesus monkeys and rats may be explained by the higher capacity of the rat adrenal glands to synthesize and release progesterone compared to the primate adrenal glands [68]. Thus, administration of interleukin in rats may induce the release of adrenal progesterone at concentrations that become inhibitory to LH secretion, whereas in rhesus monkeys the activation of the HPA axis by interleukin administration may result in the release of adrenal progesterone at concentrations equivalent to those observed during the preovulatory midcycle rise (cited by Xiao and Ferin [68]).

#### Women

The surgical stress of bilateral ovariectomy plus total hysterectomy performed under general anesthesia during the mid- to late-follicular phase of the menstrual cycle induces a small increase in plasma levels of LH 36 h after surgery (from ≈ 6 IU/L to 8 IU/L), preceded by an elevation of adrenal progesterone [43]. Likewise, women undergoing cholecystectomy under general anesthesia during the early- to mid-follicular phase exhibit a small but significant increase in plasma levels of LH (from ≈ 6 IU/L to 12 IU/L) and FSH (from ≈ 6 IU/L to 9 IU/L) 12 h after the operation, coinciding with a peak in plasma progesterone [42]. However, no wave-like changes in plasma LH levels are observed in women undergoing bilateral ovariectomy plus total hysterectomy during the early- to mid-luteal phase [43].

Although some studies report that light, heavy or exhaustive exercise does not alter [69] or even decreases [46] plasma levels of LH at the mid-follicular or mid-luteal phases, other studies evidence small increases in plasma levels of LH in response to acute physical exercise. In fact, 60 min of progressive submaximal treadmill exercise in the mid-follicular phase produces a small surge-like increase in plasma levels of LH (from ≈ 10 to 14 IU/L) in 24-h fasted eumenorrheic women [49] as well as a significant stimulatory effect on maximal peak amplitude of LH pulses (from 8.8 to 9.5 IU/L) in sedentary eumenorrheic women [53]. In contrast, the same stress applied in the mid-luteal phase [70] does not affect the LH pulse characteristics.

#### Concluding remarks

The studies analyzed in this section show conflicting results on the effects of ovarian cycle on acute-stress-induced LH release in rats. Some studies seem to indicate that the adenohypophysis is much more responsive to acute stressors on the morning of proestrus than at diestrus. In contrast, other studies suggest the opposite. It can be hypothesized that the degree of adrenal-progesterone response depends on the strength of the stressor applied. Thus, strong stressors such as intracerebroventricular injection of interleukin-1α or β or rapid blood volume depletion may induce an adrenal-progesterone response higher than that elicited by more moderate stressors such as laparotomy. Such an adrenal-progesterone response would inhibit the release of LH if plasma levels of estradiol are elevated (proestrus morning) but it would stimulate the release of LH under low estradiol backgrounds (e.g. the diestrus stage). Likewise, a moderate rise in adrenal-progesterone concentrations equivalent to that observed during the preovulatory midcycle rise would be stimulatory if it takes place under high plasma levels of estradiol (proestrus morning) but it would be unable to elicit a release of LH under low estradiol backgrounds (e.g. the diestrus stage).

In rhesus monkeys, females at the mid-follicular and mid-luteal phases, but not at the early-follicular stage, display appropriate plasma levels of estradiol for acute stressors to induce a release of LH.

Although there are some conflicting reports in women, it seems that the mid-follicular phase gathers more favorable conditions than the mid-luteal phase for the release LH by the adenohypophysis in response to acute stressors.

### Effect of hormone treatment

Table 4 shows the effect of hormone treatment on acute-stress-induced LH release in rats, rhesus monkeys and women.

**Table 4:** Effect of hormone treatment on acute-stress-induced LH release.

Species	Stressor	Hormone treatment	LH release	References
Rats	1-h or 10-min immobilization	Estradiol vs no hormone treatment	Positive	[71,72]
	Rapid blood volume depletion	Estradiol+progesterone+thyroxine vs no hormone treatment	Positive	[66]
	Transfer to a novel environment followed by a return to the original quarters 30 min later, or 15-min exposure to strobe light	No hormone treatment vs estradiol	Positive	[72]
Rhesus monkeys	30-min intracerebroventricular infusion of interleukin-1α	Estradiol at doses that result in plasma estradiol levels that reproduce the late follicular phase	Positive	[56]
		Estradiol at doses that result in plasma estradiol levels that reproduce the early-mid follicular phase	No effect	[57]
Women	Single intravenous injection of endotoxin	Estradiol vs no hormone treatment	Positive	[35]

#### Rats

In contrast to ovariectomized non-estrogen-primed rats, ovariectomized estrogen-primed rats respond with a small but significant LH peak (≈ 0.017 IU/L) either 1 h after the onset of a prolonged (11 h) immobilization [71] or 10 min after a short (15 min) immobilization [71]. Likewise, ovariectomized rats treated with estradiol, progesterone and thyroxine respond with an LH surge (zenith: ≈ 0.052 IU/L) 10 min after undergoing a rapid blood volume depletion from the external jugular veins. This response, however, is not observed in ovariectomized non-treated rats [66]. We should note, however, that acute stressors such as the transfer of rats to a novel environment followed by a return to their original quarters 30 min later, or 15-min exposure to a strobe light have no effects on serum LH in 2-week ovariectomized estradiol-primed rats, but significantly enhance LH release (zenith: ≈ 0.015 IU/L and ≈ 0.020 IU/L, respectively) in 2-week ovariectomized non-estrogen-primed rats [72].

#### Non-human primates

In ovariectomized rhesus monkeys after 5 days of estrogen replacement therapy at doses simulating the late follicular phase, a 30-min intracerebroventricular infusion of interleukin-1α results in a small but significant increase in LH release during the first 5 h after treatment (from 0.014 IU/L to 0.019 IU/L) [56]. However, the response of LH is prevented in replaced monkeys with plasma levels similar to those of the early-mid follicular phase [57].

#### Women

Estrogen-treated menopausal or ovariectomized women respond to an inflammatory/immune-like stress, induced by single intravenous injection of endotoxin, with an ovulatory-like LH peak (50 IU/L) 7 h after injection. In contrast, non-estrogen treated women do not modify their basal LH levels after endotoxin injection [35].

#### Concluding remarks

Studies in ovariectomized rats show paradoxical results as far as the response of the adenohypophysis to different acute stressors is concerned. Whereas 2 studies [66,71] found a stimulatory effect of estradiol replacement on acute-stress-induced LH release, Briski and Sylvester [72] evidenced that several types of acute stress exerted different effects on pituitary LH release and that the steroid environment modulated in a different way (inhibiting or stimulating) the pattern of response of the HPG axis induced by these stressors.

Literature focused on rhesus monkeys shows a stimulatory effect of estradiol replacement on acute-stress-induced LH release at doses that result in plasma estradiol levels reproducing the late follicular phase. This stimulatory effect, however, is not found after estradiol treatment at doses that result in plasma estradiol levels simulating the early-mid follicular phase.

Finally, estrogen-treated menopausal or ovariectomized women respond with a clear ovulatory-like LH peak after a single intravenous injection of endotoxin [35].

## Summary conclusions

The studies reviewed in this bioessay indicate that the ovarian cycle and, in particular, the female's estradiol background modulates the response of the HPA and HPG axes to acute stressors. The pattern of response, however, differs between the HPA and HPG axes. In fact, although the highest responses of the HPA axis within the ovarian cycle are observed when females display relatively high plasma levels of estradiol (proestrus morning in rats, mid-follicular phase in rhesus monkeys and mid-follicular and mid- and late-luteal phases in women), the HPA axis exhibits positive responses in practically all phases of the ovarian cycle. In contrast, it seems that there is only one specific period of time within the ovarian cycle during which the HPG axis response is possible. In particular, positive responses of the HPG axis are found under relatively high plasma levels of estradiol on the morning of proestrus in rats, during the mid-follicular and mid-luteal phase in rhesus monkeys and during the mid-follicular phase in women. This conclusion is endorsed by the fact that the HPG axis of estrogen-treated ovariectomized females and perimenopausal or menopausal women also exhibit a positive response to acute stressors.

Two studies [66,72] in the rat show that several types of acute stress may exert different effects on pituitary LH release and that the steroid environment may modulate in a different way (inhibiting or stimulating) the pattern of response of the HPG axis induced by acute stressors. In rhesus monkeys and women, the steroid environment may also modulate in a different way the pattern of response of the HPA axis (and therefore the response of HPG axis including release of LH) elicited by acute stressors. In particular, in estrogen-treated ovariectomized females and perimenopausal or menopausal women, some ranges of plasma estradiol concentration seem to prevent whereas others allow a response of the HPA axis to acute stressors.

In women, the pattern of LH release elicited by acute stressors may vary from small non-ovulatory rises in plasma concentrations and/or slight changes in pulse characteristics to the typical ovulatory surge that spontaneously takes place at the middle of the menstrual cycle. As women present waves of follicular development during an interovulatory interval (and likely during pregnancy and lactation), they may be induced to ovulate at any point of the menstrual cycle or even during periods of amenorrhea associated with pregnancy and lactation if exposed to an appropriate acute stressor under a right estradiol environment. We should bear in mind that the maximum diameters (range of 10-17 mm) of the dominant follicles from anovulatory major waves, which account for 21% of the anovulatory follicular waves, are compatible with potential ovulations if follicles were correctly stimulated by an ovulatory-like LH surge. Follicles large enough (≥ 15 mm of diameter) to ovulate may be found in the ovaries of all the regular-cycling women at the late-mid follicular phase (growing antral follicles from the ovulatory follicular wave). In addition, they may be present in the ovaries of women exhibiting (1) a major-major 2-wave pattern at the mid-luteal phase (10% of the overall population of regular-cycling women); (2) a minor-major-major 3-wave pattern at the early-mid-follicular phase (6% of the overall population of regular-cycling women); and (3) a major-major-major 3-wave pattern at the late-mid-luteal and early-mid follicular phases (6% of the overall population of regular-cycling women) [13]. We should note, however, that ovulation during the luteal phase or during pregnancy and lactation are extremely rare due to the presence of negative feedbacks that prevent the LH surge.

We should bear in mind that an acute-stress-induced surge of LH is shortly preceded by an elevation of serum progesterone from the adrenal glands. This fact suggests that such an elevation of serum progesterone may advance the secretory transformation of the endometrium resulting in embryo-endometrium asynchrony and consequently reduced chances of implantation and pregnancy if ovulation and fertilization took place. However, a recent systematic review and meta-analysis [73] has not detected detrimental effects of elevated levels of progesterone on the day of human chorionic gonadotropin (hCG) administration on the probability of clinical pregnancy in women undergoing ovarian stimulation with GnRH analogues and gonadotropins for in vitro fertilization. Although this may not be the case for women treated with GnRH antagonists (for review, see Venetis et al. [73]), we should take into account that an elevation of plasma progesterone on the day of hCG administration should be sustained in order to impair endometrial receptivity. This is not the case in estrogen-treated menopausal or ovariectomized women after being exposed to single intravenous injection of endotoxin [35]. These women display a wave-like adrenal release of progesterone. In particular, plasma progesterone concentrations rise from ≈ 0.2 nmol/L 1-2 h after injection to ≈ 5 nmol/L 4-5 h after injection. Thereafter, plasma levels of progesterone drop to reach their baseline levels of ≈ 0.2 nmol/L 7 h after injection.

## Competing interests

The authors declare that they have no competing interests.

## Authors' contributions

JJT has been involved in conception and design, acquisition, analysis and interpretation of data and drafting the article. TH and AC have been involved in analysis and interpretation of data, and revising the article critically for important intellectual content. All authors read and approved the final manuscript.
